# Arctic Edible Brown Alga *Fucus distichus* L.: Biochemical Composition, Antiradical Potential and Human Health Risk

**DOI:** 10.3390/plants12122380

**Published:** 2023-06-19

**Authors:** Ekaterina D. Obluchinskaya, Olga N. Pozharitskaya, Elena V. Gorshenina, Denis V. Zakharov, Elena V. Flisyuk, Inna I. Terninko, Yuliya E. Generalova, Alexander N. Shikov

**Affiliations:** 1 Murmansk Marine Biological Institute of the Russian Academy of Sciences (MMBI RAS), 17 Vladimirskaya Str., 183038 Murmansk, Russia; obluchinskaya@gmail.com (E.D.O.); zakharden@yandex.ru (D.V.Z.); 2 Zoological Institute RAS (ZIN RAS), 1 Universitetskaya Embankment, 199034 Saint-Petersburg, Russia; 3 Department of Technology of Pharmaceutical Formulations, St. Petersburg State Chemical Pharmaceutical University, 14 Prof. Popov Str., 197376 Saint-Petersburg, Russia; elena.flisyuk@pharminnotech.com; 4 Core Shared Research Facilities “Analytical Center”, St. Petersburg State Chemical Pharmaceutical University, 14 Prof. Popov Str., 197376 Saint-Petersburg, Russia; inna.terninko@pharminnotech.com (I.I.T.); Generalova.Yuliya@pharminnotech.com (Y.E.G.)

**Keywords:** arctic, *Fucus distichus*, carbohydrates, polyphenols, algae, toxic metals, antioxidants

## Abstract

*Fucus distichus* L. is the dominant canopy-forming macroalga in the rocky intertidal areas of the Arctic and Subarctic. In the present study, the impact of the geographic location of *F. distichus* collected in the Baffin Sea (BfS), Norwegian Sea (NS), White Sea (WS), and Barents Sea (BS) on the variations in biochemical composition, antiradical properties, and health risk was evaluated. The accumulation of main carbohydrates (fucoidan, mannitol, and alginic acid) varied from 335 mg/g dry weight (DW) in NS to 445 mg/g DW in BS. The highest level of the sum of polyphenols and flavonoids was found in samples of *F. distichus* from WS and was located in the following ranking order: BS < BfS < NS < WS. The 2,2-diphenyl-1-picrylhydrazyl radical scavenging activity of seaweed is correlated with its phenolic content. It is notable that in most Arctic *F. distichus* samples, Cd, Cr, Pb, and Ni were not detected or their concentrations were below the limit of quantification. According to calculated targeted hazard quotient and hazard index values, all studied samples of Arctic *F. distichus* are safe for daily consumption as they do not pose a carcinogenic risk to the health of adults or children. The results of this study support the rationale for using Arctic *F. distichus* as a rich source of polysaccharides, polyphenols, and flavonoids with important antiradical activity. We believe that our data will help to effectively use the potential of *F. distichus* and expand the use of this algae as a promising and safe raw material for the food and pharmaceutical industries.

## 1. Introduction

Arctic brown seaweeds are a specific source of unique compounds that may be used to create various products with beneficial properties. In the Northern Hemisphere, the intertidal areas of many cold and warm temperate regions are dominated by the genera Fucus, Ascophyllum, and Pelvetia of the Fucaceae family [1,2]. These algae are the most prominent and have increased relevance due to their high content of various phytochemicals with industrial applications [3]. The dominant canopy-forming macroalga in the rocky intertidal areas of the Arctic and Subarctic is *Fucus distichus* Linnaeus, 1767 [2,4]. The dynamic development and almost ubiquitous distribution of *F. distichus* in the littoral zone of the shelf allow us to consider this species as a potential commercial one. In an undisturbed natural environment, the biomass of *F. distichus* can reach 25 kg/m^2^ [5]. The chemical composition and high ecological and economic value of Fucus spp. stimulate significant interest and promote the study of its chemical composition and activities for practical applications. *F. distichus* is nutritionally rich macroalga, containing, based on dry weight, 8.1–10.0% of protein, 1.1–3.0% of lipids, 17.6–26.7% of soluble carbohydrates, 70.6% of total carbohydrate content, and 18.6–20.5% of minerals [6,7,8,9]. However, these values have geographical and seasonal variations [7].

Like other Phaeophyceae, *F. distichus* is a rich source of valuable biologically active compounds such as fucoidans [8,10,11], mannitol [8,12], alginic acid [8,12], pigments [13], phenolic constituents, and essential minerals [14,15]. Fucoidans are common in the Fucaceae family and are only present in brown seaweeds [16,17]. Fucoidan from *F. distichus* is composed of 61.9 mol.% fucose, 6.9% sulfate, and 26.1% uronic acid [10]. The main structural units are represented by 1→ 4 and 1→ 3 linked *L*-fucose [12,18,19]. *F. distichus* fucoidan exhibits anti-inflammatory and anticoagulant activities [10], which have a beneficial effect on age-related macular degeneration [20,21]. In previous publications, the promising antioxidant potential of *F. distichus* was associated with I ts high phlorotannin content [11]. Brown seaweeds, like *F. distichus* are known for their enhanced capacity for the accumulation of minerals and organic and inorganic contaminants from sediments and seawater due to their unique structural and physiological characteristics [22,23,24,25]. Consuming edible seaweed regularly could result in increased health hazards due to its capacity to accumulate elements.

In the present study, the biochemical composition of *F. distichus* L. collected in different seas of the Arctic region was analyzed. The antiradical properties and health risks were estimated. Our results highlight the potential of Arctic *F. distichus* as a promising source of functional compounds with multi-biological activity for use in the food and pharmaceutical industries.

## 2. Materials and Methods

### 2.1. Samples Collection

**Figure 1:**
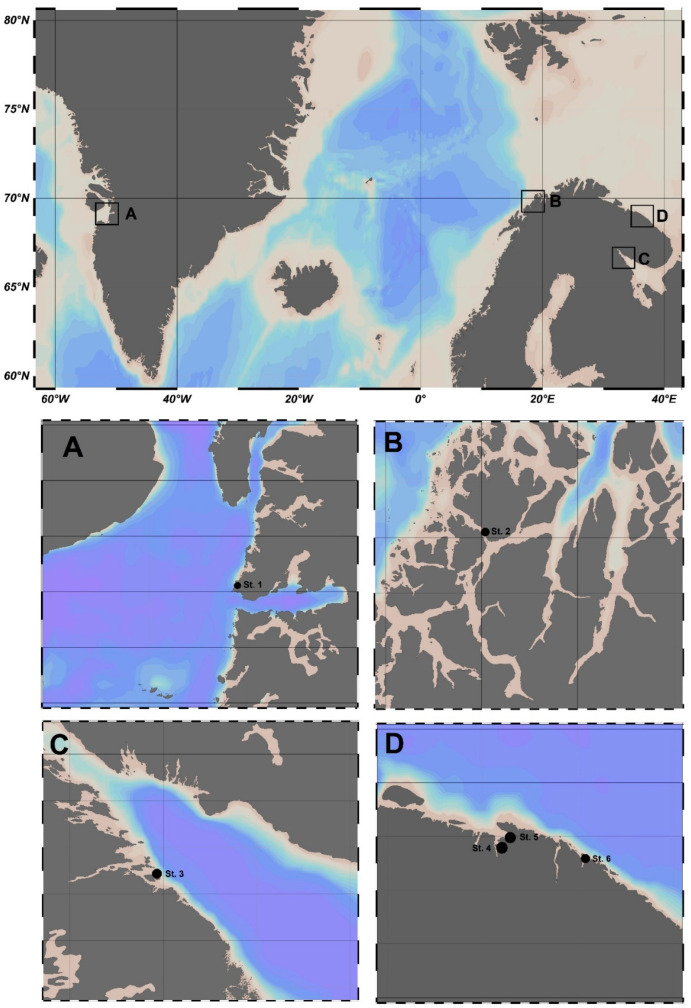
Map of the sampling sites: (**A)** Baffin Sea–Station (St.) 1 (Disko Bay); (**B**) Norwegian Sea–St. 2 (Ringvassøya Island; (**C**) White Sea–St. 3 (Pezhostrov Island of the Kandalaksha Bay); (**D**) Barents Sea–St. 4 (Teriberskaya Bay (Korabelnaya Bay)), St. 5 (Teriberskaya Bay (Zavalishina Bay)), and St. 6 (Zelenetskaya bay).

Samples of *F. distichus* were harvested in the coastal zones (low tide at 0.6–1.0 m depth) of the Baffin Sea (BfS), Norwegian Sea (NS), White Sea (WS), and Barents Sea (BS) (Figure 1) in summer 2019. The details of the collection procedure were described in [26,27].

*F. distichus* samples were collected in Greenland, Norway, and Russia, namely in: Disko Bay of the BfS (Station (St.) 1); NS, Ringvassøya Island (St. 2); WS, Pezhostrov Island of the Kandalaksha Bay Islands (St. 3); and BS, Teriberskaya and Zelenetskaya bays (St. 4–6) (Table 1).

### 2.2. Chemicals

DPPH (2,2-diphenyl-1-picrylhydrazyl), quercetin, phloroglucinol, fucose (>99%), xylose (>99%), and the Folin-Ciocalteu reagent were all purchased from Sigma-Aldrich (St. Louis, MO, USA). Local chemical suppliers provided all other analytical-grade chemicals and solvents for extraction and testing. Ultrapure water (resistivity of 18.2 MΩ cm) for all solution preparations was obtained using a Milli-Q purification system (Millipore, Bedford, MA, USA). Multi-Element Calibration Standard 3 for element analysis was from PerkinElmer, USA.

### 2.3. Carbohydrates Composition

For fucoidan content determination, seaweed samples were processed following the procedure described in [28]. The fucoidan content was measured by the cysteine-sulfuric acid method [29]. *L*-fucose was used as a reference.

For carbohydrate analysis, fucoidan samples (10–15 mg) were hydrolyzed with 2 M trifluoroacetic acid (0.5 mL) at 121 °C for two hours to determine the concentration of monosaccharides. Then samples were cooled in an ice water bath, centrifuged at 5000 rpm for 5 min, and the liquid fraction was adjusted to pH 7 with 2 M NaOH [30]. The carbohydrate content was estimated by high-performance liquid chromatography (HPLC Model LC 20 AT Prominence, Shimadzu, Kyoto, Japan) and equipped with a refractive index detector (RID-10A, Shimadzu, Kyoto, Japan) as described previously [31].

The level of mannitol in *F. distichus* samples was determined according to E. Obluchinskaya (2008). Briefly, the powdered seaweed sample (3 g) was extracted three times with 25 mL aqueous solution of CuSO_4_ (0.5% *w*/*v*) in a boiling water bath for 0.5 h. Afterward, the mixture was filtered, combined, and made up to 100 mL with water. 10 mL sample solution was mixed with 0.1 mL concentrated H_2_SO_4_ and after 30 min added 5 mL 4 M NaOH and 5 mL CuSO_4_ (12.5% *w*/*v*). The solutions were mixed and incubated for one hour at room temperature. Centrifugation was used to remove the precipitate. Finally, the absorbance was measured at 597 nm (Shimadzu UV 1800, Shimadzu, Kyoto, Japan) and compared to a mannitol calibration curve [32]. Results were expressed in percent per dry weight (DW). All measurements were performed in triplicate.

The alginic acid content was determined by reaction with 3,5-dimethylphenol and sulfuric acid [33]. Briefly, 0.5 mL of alginic acid (ranging from 0.002 to 0.1 mg/mL) and 0.5 mL 0.01% water solution of the sample was added to 0.5 mL of 20% H_3_BO_3_ in a 0.1 M NaOH solution and 4 mL of concentrated H_2_SO_4_. The solutions were mixed and incubated at 22 °C for 10 min and then at 70 °C in a water bath for 40 min, and after cooling to room temperature, 0.1 mL 3.5-dimethylphenol (0.1% *w*/*v*) was added and stored for 180 min at room temperature. The absorbance of the standards and extracts was measured at 400 nm (A400) and 450 nm (A450). Alginic acid was used as a standard at an absorbance A400–A450. Results were expressed as percentages per DW, and all measurements were conducted three times for accuracy.

### 2.4. Analysis of Total Phenolic, Total Flavonoids, and Antiradical Activity

For the analysis of the content of total phenolics (TPC), total flavonoids (TFC), and DPPH scavenging activity of the samples of *F. distichus,* they were extracted by the method [34] with some modifications. Briefly, the powdered seaweed samples (2 g) were extracted three times with 50 mL aqueous MeOH (60% *v*/*v*) in a dark place at room temperature for 24 h under continuous stirring at 200 rpm on a Multi Bio RS-24 rotator (Biosan, Riga, Latvia). Afterward, the mixtures were centrifuged at 3500 rpm for 10 min, filtered (Whatman filter paper N 1), and combined. The filtrate was concentrated to dryness under reduced pressure using a rotary evaporator IR-1m (PJSC Khimlaborpribor, Klin, Russia) to remove MeOH, and the residue was dissolved in 25 mL volumetric flasks with 60% (*v*/*v*) aqueous MeOH and filtered before use for analysis of the TPC, TFC, and DPPH scavenging activity. Extraction assays were performed in triplicate.

The TPC in the *F. distichus* extracts was analyzed spectrophotometrically at 750 nm (Shimadzu UV 1800 spectrophotometer, Shimadzu, Kyoto, Japan) according to [35] using the Folin-Ciocalteu reagent. TPC was expressed as mg phloroglucinol equivalent (PhE) per g DW.

The TFC was measured by a spectrophotometric assay [34,36], with some modifications [37]. The absorbance of the tested solutions was recorded at 415 nm on a UV-Vis spectrophotometer, Shimadzu UV 1800 (Shimadzu, Kyoto, Japan). TFC was expressed as mg quercetin equivalent (QE) per g DW.

The DPPH scavenging activity was analyzed according to W. Brand-Williams et al. [38], with some modifications [37]. The absorbance of the resulted solutions was measured at 517 nm with the UV-Vis spectrophotometer Shimadzu UV 1800 (Shimadzu, Kyoto, Japan).

The percent DPPH scavenged by each different samples was calculated according to Equation (1):

(1) $$DPPH\;scavenging\;activity\;(\%)=\frac{A_{control}-A_{sample}}{A_{control}}\times 100$$

where *A_control_* stands for the absorbance of the control, and *A_sample_* is the absorbance of the sample solution reaction at 30 min.

The percentage of remaining DPPH-radicals was plotted against the sample/standard concentration to obtain the IC_50_ value, which represents the concentration of the extract or reference antioxidant (mg/mL) required to scavenge 50% of the DPPH-radicals in the reaction mixture. Its reciprocal, the antiradical power (ARP, ARP = 1/IC_50_), was also calculated for each of the sample extracts [39].

All measurements were performed in triplicate.

### 2.5. Element Analysis

The samples of *F. distichus* were extracted by the method [35]. The PerkinElmer^®^ Optima™ 8000 inductively coupled plasma optic emission spectrophotometer (ICP-OES) (PerkinElmer, Inc., Shelton, CT, USA) was used for the analysis of elements as described previously [37]. Instrumental parameters were as described by É. Flores et al. [40]. The concentration of the elements (mg/kg) was calculated according to Equation (2):

(2) $$X=\frac{C_{calib}\times V\times 1000}{m}$$

where *C_calib_*—element concentration from the calibration, mg/L; *V*—volumetric flask, L; *m*—sample weight, g.

For the evaluation of the accuracy of the method, a reference sample of Cu was added to the *F. distichus* sample as described in [41]. The mean recovery value of Cu was 94–104%.

### 2.6. Metal Pollution Index

The metal pollution index (MPI) [42] represents the contribution of all the elements detected and calculated according to Equation (3):

(3) $$MPI={\left(M_{1}\times M_{2}\times \ldots \times M_{n}\right)}^{1/n}$$

where *Mn* is the concentration of the metal *n* in the sample in mg/kg.

### 2.7. Assessments of Human Health Risk

The nutritional recommendations [43,44] were used for the evaluation of the nutrimental importance of essential elements. The health risk associated with the toxic elements accumulated by *F. distichus* samples was assessed using risk estimators [43,44,45,46,47].

The risk to human health from the elements contained in *F. distichus* samples was assessed using the targeted hazard quotient (THQ) and hazard index (HI) proposed by USEPA (2020). The indexes were calculated following Equations (4)–(6) below [48,49].

(4) $$Estimated\;Daily\;Intake\;\left(EDI\right)=\frac{Ci\times CR\times EF\times ED}{BW\times AT}$$

(5) $$Target\;Hazard\;Quotient\;\left(THQ\right)=\frac{EDI}{RfD}$$

(6) $$Hazard\;Index\;\left(HI\right)=\sum_{k=1}^{n=k}THQ$$

where *Ci* is the mean concentration of each element in the sample (mg/kg); *CR* is the consumption rate (0.0052 kg); *EF* is the exposure frequency (250 days); *ED* is the average exposure duration (70 years); *BW* is the average body weight (70 kg) and *AT* is the average lifetime (72.59 years) [50]. There is no fixed consumption rate for seaweed in Russia. As a result, the consumption rate has been considered in different studies [49]. *RfD* is the recommended oral reference dose.

### 2.8. Statistical Analysis

The statistical analysis was conducted using STATGRAPHICS Centurion XV (StatPoint Technologies Inc., Warrenton, VA, USA). Data and error bars in the figures are expressed as mean ± standard deviation (SD). Differences between means were analyzed by the ANOVA test, followed by the post hoc Tukey’s test. The difference was considered significant at a level of *p* < 0.05. Pearson’s correlation coefficients were used to establish the relationship between the content of representative compounds and antioxidant capacity. Multiple regression and multivariate data analysis using the partial least squares coefficient method was carried out.

## 3. Results and Discussion

### 3.1. Carbohydrates Composition

Literature data on the study of the carbohydrate composition of *F. distichus* are very insufficient, and mainly fucoidan has been studied. However, the content of fucoidan in samples collected in different seas of the Arctic was compared for the first time.

**Figure 2:**
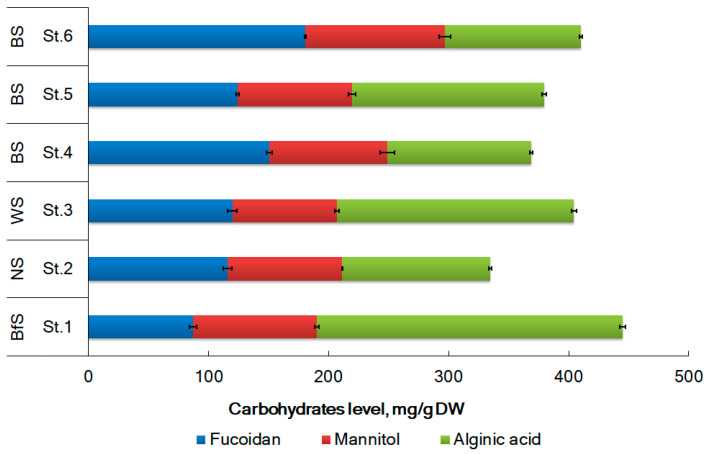
The level of main carbohydrates from *F. distichus* from different seas of the Arctic region. BfS (Baffin Sea)–St. 1 (Disko Bay), NS (Norwegian Sea)–St. 2 (Ringvassøya Island), WS (White Sea)–St. 3 (Pezhostrov Island), and BS (Barents Sea)–St. 4 (Korabelnaya Bay), St. 5 (Zavalishina Bay), and St. 6 (Zelenetskaya bay) (error bars for SD at n = 3). The station locations (St. 1–St. 6) are presented in Figure 1.

The content of fucoidan in the tested samples of *F. distichus* varied from 86.9 ± 3 mg/g DW from the Disco Bay of the BfS to 180.6 ± 0.8 mg/g DW in the Zelenetskaya Bay of the BS (St. 6) (Figure 2). The content of fucoidan in *F. distichus* samples from the WS and the NS was 116.1 ± 3.6 and 119.8 ± 3.7 mg/g DW, respectively, and there is not a statistically significant difference between the standard deviations of the two samples at the 95.0% confidence level. For Teriberskaya Bay in BS, values varied from 150.5 ± 2.6 (St. 4) to 124.3 ± 1.0 mg/g DW (St. 5). The accumulation of fucoidan is not affected by water temperature or salinity. The level of fucoidan from *F. distichus* collected in autumn in Zelenetskaya Bay was slightly lower and reached 146.7 ± 22.4 mg/g DW. It is interesting to note that T.N. Zvyagintseva et al. (2003), having analyzed samples of some Far Eastern brown algae, also found some noticeable differences for samples obtained in different geographical locations [51].

The levels of fucose and xylose in *F. distichus* from different locations determined by HPLC-RID after acid hydrolysis are presented in Table 2.

**Figure 3:**
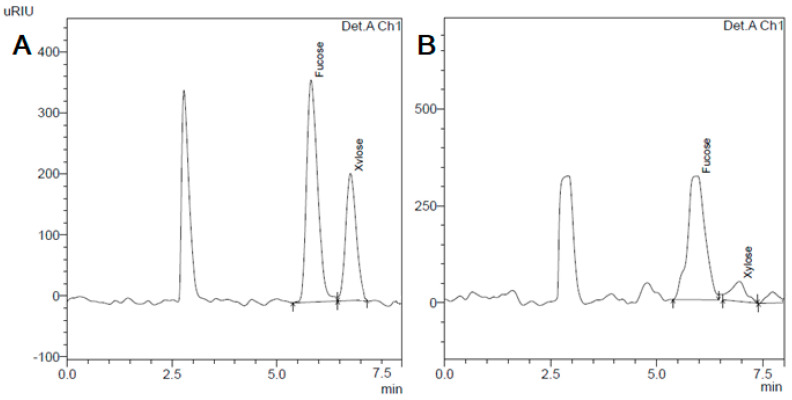
The typical chromatogram of (**A**) reference compounds fucose and xylose and (**B**) a sample of *F. distichus* from the BS (Barents Sea), St. 4 (Korabelnaya Bay).

The content of the main monosaccharides, determined by HPLC after acid hydrolysis, fucose, in the samples ranged from 43.5 mg/g in BfS (St. 1) to 90.3 mg/g in BS (St. 6). Previously, it was found that fucose is dominant in these seaweed species. Its content varied from 59.4–62 mol.% [11,52,53] to 76.7–87 mol.% [54,55]. Xylose (4.5–10 mol.%) was a minor sugar. The content of galactose, mannose, and glucose was less than 7 mol.% or a trace [17]. In this study, samples from the Barents Sea were most rich in fucose and xylose (Table 2). The typical chromatogram is presented in Figure 3.

A strong correlation between accumulation of fucose and xylose (Pearson’s correlation coefficients *r* = 0.926, *p* < 0.05) and their ratio (*r* = −0.682 and *r* = −0.887, *p* < 0.05 for fucose and xylose, respectively) was established. No correlation was observed between xylose and fucose contents and water salinity, while a slight negative correlation between water temperature and fucose and xylose content (Pearson’s correlation coefficients *r* = −0.378 and *r* = −0.465, *p* < 0.05, respectively for fucose and xylose) was found. The fucose/xylose ratio in fucoidan from the samples collected in the BfS differed significantly from the rest of the samples and was 1.45 times higher on average.

Crude fucoidan from *F. distichus* harvested in the Kiel Fjord (Germany) contained about 61.9−76.7 mol.% fucose, and the fucose to xylose ratio was 6.13−7.83 [20,54]. Fucoidan extracted from *F. distichus* from the western coast of Iturup Island (the Okhotsk Sea) collected in summer was composed of 59.4 mol.% fucose and 5.7 mol.% xylose, and the ratio of fucose to xylose was 10.42 [52]. While purified fucoidan contained 87.1 mol.% fucose and an increased ratio of fucose to xylose of 24.19 [56], T.N. Zvyagintseva et al., (2003) have found some notable differences for the *F. distichus* collected in different spots of the southern Okhotsk Sea (fucose proportion 56–80%) [51]. Previously, A.V. Skriptsova et al., (2012) showed that the fraction of fucose changes insignificantly during the transition to the generative phase. Based on the obtained data and literature, it can be assumed that the predominant unit of fucoidan synthesized by *F. distichus* is fucose [57].

Mannitol content in the tested samples ranged from 86.9 to 116.2 mg/g DW (Figure 2). Mannitol levels were statistically higher in samples from the BfS (St. 1) and BS (St. 6) than in samples from the WS (St. 3) (103.0 ± 1.8 mg/g DW and 116.2 ± 4.6 mg/g DW compared to 86.9 ± 1.8 mg/g DW, *p* < 0.05). A positive correlation between the content of mannitol in algae and the water salinity was found (Pearson’s correlation coefficients, *r* = 0.41, *p* < 0.05).

Hexatomic alcohol *D*-mannitol is one of the primary products of photosynthesis and a reserve substance in brown algae. Its content is varied by different seaweeds, seasons, and growing conditions. Mannitol has various technical and medical applications, and its isolation from algae is cheaper than chemical synthesis [58]. The richest sources of *D*-mannitol are representatives of the genus Laminaria, and they can accumulate up to 20–30% of the dry weight of biomass. The content of mannitol in the biomass of the focus algae from Kamchatka was about 7.7% DW [59]. *F. distichus* from the Zelenetskaya Bay of the Barents Sea accumulates mannitol up to 12.75% DW [32]. The positive correlation between the water salinity and the mannitol content in *F. vesiculosus* has been demonstrated [60]. It supports its osmoregulatory functions in brown seaweeds.

The content of alginic acid in the tested samples of *F. distichus* varied from 113.2 ± 1.1 mg/g DW from the Zelenetskaya Bay of the Barents Sea (St. 6) to 255.1 ± 2.4 mg/g DW in the Disco Bay of the Baffin Sea (St. 1) (Figure 2). For Teriberskaya Bay in the Barents Sea, the values varied from 119.7 ± 1.4 (St. 4) to 159.8 ± 1.8 mg/g DW (St. 5). No correlation was found between temperature, salinity, and the content of alginic acid. The level of alginic acid from *F. distichus* collected in autumn in Zelenetskaya Bay was slightly lower and reached 235.8±24.0 mg/g DW [32]. According to reference [28], the content of alginic acid found in Fucus collected from Avacha Bay in Kamchatka was 173 mg/g DW.

Several therapeutic activities, such as anticoagulants, antitumor agents, and others, have been demonstrated for alginate in vivo [61]. Due to their lack of toxicity and adaptation to demands, alginate polymers have significant potential for the development of pharmaceutical, biomedical, and food formulations. Some alginate-containing gastrointestinal formulations and protectors (e.g., Gaviscon) have been reported in the literature [62].

### 3.2. Polyphenols and Flavonoids Content

**Figure 4:**
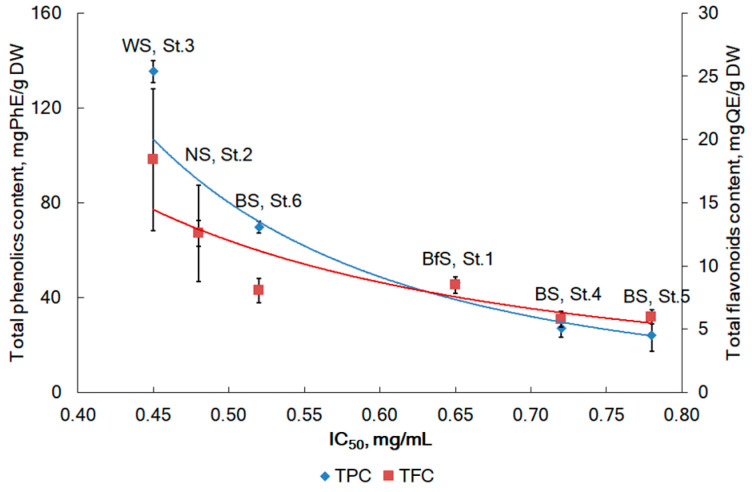
The correlation between DPPH scavenging activity (expressed as IC_50_) and TPC and TFC in *F. distichus* collected in different Arctic seas: experimental data—markers (error bars for SD at n = 3), and the correlation—lines (blue—TPC; red—TFC). BfS (Baffin Sea)–St. 1 (Disko Bay), (NS) Norwegian Sea–St. 2 (Ringvassøya Island), WS (White Sea)–St. 3 (Pezhostrov Island), and BS (Barents Sea)–St. 4 (Korabelnaya Bay), St. 5 (Zavalishina Bay), and St. 6 (Zelenetskaya bay) (error bars for SD at n = 3). The station locations (St. 1–St. 6) are presented in Figure 1.

The TPC in *F. distichus* collected in different Arctic regions varied in a wide diapason of concentrations, from 24.0 to 135.3 mg of phloroglucinol equivalent per 1 g of DW. The TFC was on average 6.2 times lower than the TPC (Figure 4).

The highest accumulation of TPC and TFC was observed in samples of *F. distichus* from the White Sea and was increased in the following ranking order: BS_mean_ < BfS < NS < WS.

Brown algae synthesize phlorotannins, polyphenolic compounds that include phloroglucinol units in their structure [63]. The extract from algae of the order Fucales was the most distinct in phlorotannin content compared to the order [14]. According to the previous publication, the antioxidant activity of fucoidan from the brown alga was associated with the impurity of phenolic compounds [64].

### 3.3. DPPH Radical Scavenging Activity

The DPPH radical scavenging activity of *F. distichus* was expressed as antiradical power (ARP), which represents the reciprocal of IC_50_ (ARP = 1/ IC_50_). All the investigated samples of *F. distichus* exhibited medium or low activity in the DPPH assay. The ARPs ranged from 1.3–1.4 in BS to 2.2 in WS (Figure 3). The sample from BS (St. 5) had the lowest polyphenol content (24.4 mg/g DW) and the lowest ARP of 1.2 mL/mg. In contrast, the sample from WS showed strong DPPH radical scavenging activity with an ARP value of 2.2 (Figure 3). A similar scavenging activity pattern was observed for the flavonoid assay. The sample with the highest flavonoid content of 18.4 mg/g DW (WS, St. 3) showed a stronger antioxidant capacity than the other samples. In our study, a weak negative correlation was found between the content of fucose or xylose and their antiradical activity (Pearson’s correlation coefficients *r* = −0.482 and *r* = −0.359, *p* < 0.05, respectively for fucose and xylose). This finding may indicate a negative effect of fucoidan content on the radical scavenging activity of *F. distichus* extracts. Although several studies have reported that fucoidan has radical scavenging activity [65], crude fucoidan was used in the above studies. Therefore, other compounds usually observed in crude fucoidans (e.g., minor phenolics, ascorbic acid, fucoxanthin, proteins, etc.) may have an impact on the radical scavenging activity. In a previous study, only very weak antioxidant activity was found for relatively pure sulfate-rich polysaccharide fractions containing few polyphenols. T.I. Imbs et al. (2015) concluded that the structural features required for the antioxidant activity of sulfated polysaccharides from Fucus algae from the Okhotsk Sea are polyphenols co-extracted with sulfated polysaccharides [64]. The content of phenolic compounds, including phlorotannins and flavonoids, largely determined the radical-scavenging activity of samples (Pearson’s correlation coefficients r = 0.895 and r = 0.870, *p* < 0.05, for TPC and TFC, respectively).

Our results are in line with previous publications. The direct correlation between DPPH scavenging activity and TPC in algal extracts has been discussed by several authors [11,39,64]. Flavonoids contribute to the ARP too. Extreme conditions, such as salinity, dryness, air exposure, UV radiation, etc., influence on littoral algae during high and low tides. Algae synthesize a variety of chemical antioxidants, including polyphenols, in response to environmental stresses. Scientific publications confirm that compounds with antioxidant activity are produced by all classes of sea algae [66]. Besides dominating structure-forming polysaccharides, seaweeds of Fucus spp. are rich in polyphenols [39]. These marine polyphenols are highly hydroxylated, and their ARP can be up to 100 times stronger than that of polyphenols synthesized by terrestrial plants [67]. We found that temperature and salinity affect the antiradical activity of *F. distichus* from the Arctic region (Pearson’s correlation coefficients *r* = 0.636 and *r* = 0.605, *p* < 0.05, respectively for temperature and salinity). *F. spiralis* from the Portuguese coast was previously found to have high TPC levels (0.049 ± 0.005 mmol gallic acid equivalent (EGA)/g DW) [68] compared to *F. spiralis* collected in Denmark (0.044 ± 0.001 mmol EGA/g dry body weight) and much higher than in Scotland (0.014 ± 0.000 mmol EGA/g DW) [69,70]. These differences are related to geographic location and climatic differences. Higher temperatures and sun exposure in Portugal than in Scotland and Denmark caused seaweed to produce more antioxidant compounds to protect them [68].

### 3.4. Element Contents

The measured concentrations, range (minimum and maximum concentrations) for elements in each sample of *F. distichus*, and LOQ are provided in Table 3. The concentration of elements varied in the seaweed collected in different regions. Al and Fe levels in *F. distichus* from WS (St. 3) and BS (St. 6) were significantly higher than in other samples. This may be related to the dependence on photosynthetic activity, which proceeds continuously during the arctic summer [71]. The concentration of Ca averaged 14,774 mg/kg DW and reached a maximum of 25,476 mg/kg DW in the samples BS (St. 5). The Mg concentration was slightly lower and averaged about 9304 mg/kg DW. Samples from WS showed the highest concentrations of Ba, Co, Mn, and Fe. The majority of *F. distichus* samples from various Arctic regions did not show any detectable levels of Pb, Cd, Cr, or Ni, with some concentrations falling below the LOQ. Elements in seaweeds from the seas of the Arctic region can be sequenced in descending order by mean values: Ca > Mg > Sr > Fe > Al > Mn > Rb > Zn > As total > Ba > Ni > Co > Cu > Pb, Cr, Cd (< LOQ). Similar results were previously reported in the literature for the same elements in other fucales from the Arctic region [37,72].

*F. distichus* from the WS showed higher metal concentrations when compared to seaweeds collected in the NS and the BS (Table 3). Average concentrations of Cu, Cd, and Pb in fucus from BS from April 2010–2012, 2014, and 2018 were varied, such as 4–18 µg/g DW, 0.35–0.98, and 0.2–1.3 [27]. A comparison of seaweeds collected by us in the Arctic with *F. distichus* from the WS collected near the village of Rabocheostrovsk [73] showed that *F. distichus* had similar concentrations of Cu, Fe, and Zn but lower concentrations of Cd, Cr, Ni, and Pb. The increased concentration of Mn in algae samples from the WS is associated with a higher volume of river runoff into it [74]. The biogeochemical feature of the WS consists of increased background concentrations of Mn and low Cd, which are associated with the level of terigen runoff. It is assumed that the deficiency of bioavailable forms of Zn in the coastal strip of the WS, NS, and BfS is a consequence of increased biomass of macrophytes in the littoral. The levels of Fe and Mn are influenced by how close an area is to sources of terrigenous runoff. The concentration of Fe in the algae of the BfS is significantly lower than in the algae of the WS, which is associated with their adaptation to the supply of this metal from hydrothermal sources and the glacier, respectively.

The total concentration of As in the samples varied slightly and averaged 32.4 ± 15.4 mg/kg DW (Table 3). The As total contents of seaweed species belonging to Phaeophyta range from 1.89–245.19 mg/kg DW. The overwhelming majority of species of Rhodophyta, Phaeophyta, and Chlorophyta have As total contents of <30, 100 and 20 mg/kg DW, respectively. The species belonging to Phaeophyta and containing extremely high As total contents (over 100 mg/kg DW) are *Laminaria ochroleuca*, *Cystoseira barbata*, *Sargassum piluliferum*, *Hizikia fusiforme*, *F. vesiculosis*, *Laminaria digitate*, and *Melanosiphen intestinalis* [75].

Some compounds found in algae that are useful to humans also have one or more metal-binding sites. The polysaccharides in the cell walls of brown algae may have a high capacity to absorb and hold metals from the surrounding seawater [76]. Algal polysaccharides generally bind heavy metals to variable degrees. According to estimates of binding affinities, alginates (brown algae) are more likely to bind heavy metals than carrageenans (red algae) or agar (red algae) [77]. In this study, we also found a weak positive correlation between a total concentration of metals and a level of alginic acid (Pearson’s correlation coefficients *r* = 0.296, *p* < 0.05), but at the same time, we observed a negative correlation between the concentration of metals and the level of fucoidan and mannitol (Pearson’s correlation coefficients *r* = −0.493 and *r* = −0.440, *p* < 0.05 for fucoidan and mannitol, respectively).

### 3.5. Metal Pollution Index

**Figure 5:**
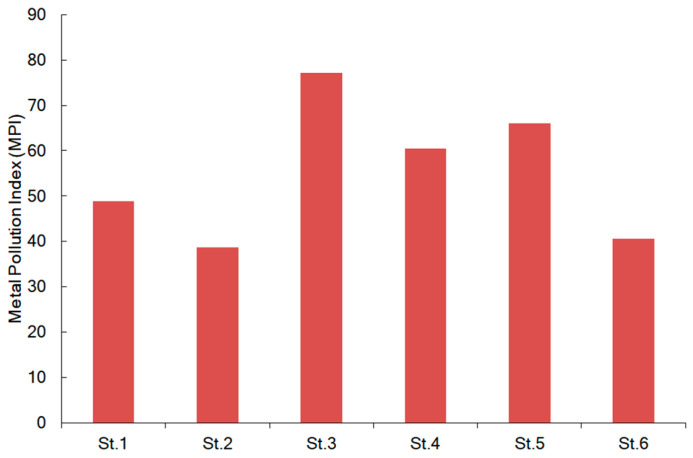
Cumulative accumulation of metals by *F. distichus* collected at different regions of the Arctic. St. 1 (Disko Bay) from BfS (Baffin Sea), St. 2 (Ringvassøya Island) from NS (Norwegian Sea), St. 3 (Pezhostrov Island) from WS (White Sea), and St. 4 (Korabelnaya Bay), St. 5 (Zavalishina Bay), and St. 6 (Zelenetskaya bay) from BS (Barents Sea). The station locations (St. 1–St. 6) are presented in Figure 1.

The cumulative accumulation of metals (MPI) by *F. distichus* collected in different regions of the Arctic is shown in Figure 5.

The overall mean MPI for all samples was 55.3 (range 39–77). Seaweeds from NS have the lowest MPI of 38.7. *F. distichus* from WS showed the highest MPI of 77.2. The MPI values in *F. distichus* have increased in the following order: NS < BfS < BSmean < WS (Figure 4). A strong Pearson correlation was found for the MPI value versus salinity (r = −0.772, *p* < 0.05) and temperature (r = 0.689, *p* < 0.05).

Various metal guidelines can be used to categorize the ecological quality of European coastal waters. According to Norwegian Pollution Control Authority guidelines for the blue mussel Mytilus edulis (SFT TA-1467/1997), different ecological classes: Unpolluted (Class I) to Very Highly Polluted (Class V) were proposed depending on metals amount [78]. Maximum metal pollutants in food were defined by the European Community Commission [79]. Previously, *F. distichus* was mentioned as a monitoring tool for seawater metal contamination [74]. Based on the data we collected on the contamination of *F. distichus* and the guidelines mentioned earlier, we can conclude that the seawater in the Arctic Region’s seas (BfS, NS, WS, and BS) in the summer of 2019 belonged to “Class I–Unpolluted” for all studied metals.

### 3.6. Human Health Risk

The mean and maximum concentration, the daily dose, and a comparison with the risk estimations for a 70 kg man [44,45,46,47] and with nutritional requirements [43,44,80] are presented in Table 4 for every element detected in F. distichus.

In recent years, the consumption of seaweed has increased in Western nations. Seaweed is a splendid source of nutrition due to its high levels of protein, fatty acids, vitamins, and minerals. As a result, it’s becoming more popular to include seaweed in daily diets [61]. Approximately 35 million tons of seaweed were produced worldwide in 2019 [50]. Regulations for contaminant levels in seaweed vary across different regions of the world. The regulatory limits for selected heavy metals in seaweed food products are implemented in some countries. For example, upper limits for Pb, Cd, Sn, Hg, As, and I in seaweed for human consumption are approved in France. [76]. The limits for Pb, As, Cd, and Hg are established for algae in Russia [80]. The potential toxicity of Arctic *F. distichus* to consumers was evaluated in the present study by comparison of all tested elements with (a) the Provisional Tolerable Weekly and Monthly Intakes (PTWI and PTWM, respectively) recommended by the Joint FAO/WHO Expert Committee on Food Additives [45,46,47] and (b) the tolerable upper intake level (UL) recommended by the European Food Safety Authority [44].

After analyzing the information provided in Table 4, we have compared the intake and corresponding UL for the tested elements according to EFSA (2006). We noted that daily consumption of 3.3–12.5 g of *F. distichus* from BS (St. 5) with the highest Ca level (25.5 g/kg DW) corresponds to a daily intake of 0.18–0.32 g of this metal. This amount is equivalent to around 7.2–12.8% of the recommended daily intake for Ca, which is 2.5 g. Daily consumption of *F. distichus* (3.3–12.5 g) with the highest Cu (3.23 mg/kg DW) from BfS (St. 1) provides 0.04 mg of this metal. This level is 0.8% of the tolerated daily dose (5 mg) of Cu. The consumption of *F. distichus* from BS (St. 5) with the highest Zn (44.6 mg/kg DW) at the above-mentioned dose provides a daily intake of 0.42–0.56 mg of this element that is equal to 3.5–4.7% of the tolerable daily dose (12 mg) for Zn. The regular consumption of *F. distichus* from BS (St. 6) with the highest Al concentration (126.2 mg/kg DW) provides an intake of 0.86–1.58 mg of this metal, which is equal to about 2.2% of the tolerable daily dose (70 mg) for Al [81].

The regular consumption of *F. distichus* (3.3–12.5 g) from BS (St. 4) provides daily consumption of 0.86–1.58 mg of total As which is equal to 17–31.6% of the tolerable daily dose for total As (5 mg/day). In this study, As was analyzed as total As in *F. distichus* samples. Exposure to inorganic arsenic can have negative health effects, including an increased risk of developing diabetes [82], cardiovascular disease [83], and various types of cancer. The International Agency for Research on Cancer has classified inorganic arsenic as a human carcinogen (Group 1) [84]. It’s important to note that in marine species, As is mostly present in organic form, specifically as sugars. The trivalent (AsIII) and pentavalent (AsV) inorganic forms are toxic, but their organic derivatives (arsenopentine, arsenosugar, arsenocholine, arsenolipids, methyl arsenate, and dimethyl arsenate) have low toxicity [85]. The toxicity of arsenolipids has not been proven. Organic arsenic compounds such as arsenobetaine are classified as Group 3 (substances not classified as carcinogenic) according to the classification of the International Agency for Research on Cancer [84].

**Figure 6:**
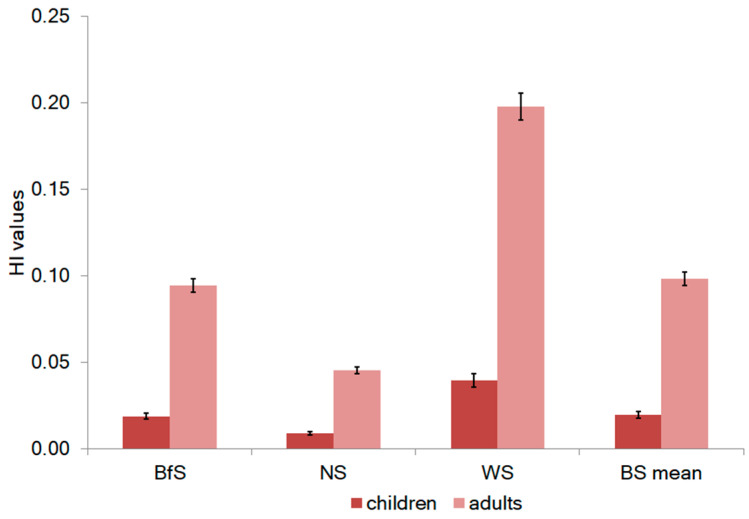
The hazard indexes (HI) for *F. distichus* collected in the Arctic (error bars for SD at n = 3). BfS (Baffin Sea), NS (Norwegian Sea), WS (White Sea), and BS (Barents Sea).

The human health risk caused by elements detected in *F. distichus* from the seas of the Arctic region was calculated for children and adults based on their daily consumption of seaweed. The targeted hazard quotient (THQ) and hazard index (HI) recommended by USEPA (2020) were used. The sum of all THQs of all elements equals HI. The calculated HI values are shown in Figure 6.

Generally, if the THQ value is below 1, there is no predicted health risk associated with some elements [86,87]. However, if the THQ is equal to or greater than 1, there may be a potential health risk that needs to be addressed through preventative or cautionary measures, as mentioned in reference [88]. In the present study, THQ for all elements in *F. distichus* samples was less than one. It indicates no potential health risk for humans. The HI values were also below one. The mean HI for all algae samples was calculated as 0.11 for adults and 0.022 for children. Thus, all studied samples of Arctic *F. distichus* are safe for daily consumption as they do not pose a carcinogenic risk to the health of adults or children (Figure 5).

It is important to note that *F. distichus,* found in the Arctic region’s seas (Baffin Sea, Norwegian Sea, White Sea, and Barents Sea), does not accumulate toxic elements in hazardous concentrations. Additionally, these algae can be used as a source of nutritional elements to meet humans’ daily nutritional needs.

## 4. Conclusions

In this study, the biochemical variability, antiradical properties, and health risks of Arctic *Fucus distichus* L. collected from the Baffin, Norwegian, White, and Barents Seas were studied. *F. distichus* from the seas of the Arctic is a rich source of carbohydrates. The accumulation of main carbohydrates (fucoidan, mannitol, and alginic acid) varied from 335 mg/g DW in NS (St. 2) to 445 mg/g DW in BS (St. 1). The highest level of the sum of polyphenols and flavonoids was found in samples of *F. distichus* from WS and was increased in the following order: BS < BfS < NS < WS. The DPPH radical scavenging activity of seaweed was correlated with its phenolic content. It is noteworthy that all the Arctic *F. distichus* samples tested did not contain detectable levels of toxic elements such as Cd, Cr, Pb, and Ni, or their concentrations were below the limit of quantification (LOQ). According to calculated THQ and HI values, samples of Arctic *F. distichus* are safe for daily consumption as they do not pose a carcinogenic risk to the health of adults or children. The results of this study support the rationale for using Arctic *F. distichus* as a valuable source of polysaccharides, polyphenols, and flavonoids. We believe that our data will help to effectively use the potential of *F. distichus* and expand the use of this alga as a promising and safe raw material for the food and pharmaceutical industries.

## Figures and Tables

**Table 1:** Characterization of collection sites of *F. distichus*.

Sea Area	Sampling Site	Coordinates	Station on Figure 1	Mean Water Temperature, °C	Range of Salinity, ‰
Baffin Sea	Disko Bay	69.219858 N 51.111819 W	St. 1	11.0	25.8–26.2
Norwegian Sea	Ringvassøya Island	69.815097 N 19.027894 E	St. 2	10.6	33.7–34.3
White Sea	Pezhostrov Island	66.273315 N 33.934406 E	St. 3	17.2	22.1–22.3
Barents Sea	Teriberskaya Bay (Korabelnaya Bay)	69.173088 N 35.168468 E	St. 4	11.2	14.7–15.5
Barents Sea	Teriberskaya Bay (Zavalishina Bay)	69.184068 N 35.259487 E	St. 5	9.1	19.9–20.7
Barents Sea	Zelenetskaya Bay	69.117150 N 36.070790 E	St. 6	10.3	31.0–32.0

St. 1–St. 6–station 1–station 6.

**Table 2:** The level of fucose and xylose in samples of *F. distichus* (mean ± SD, n = 3).

Sea, Station	Fucose, mg/g DW	Xylose, mg/g DW	Fucose/Xylose Ratio
BfS, St. 1	43.5 ± 1.5	5.4 ± 0.3	8.05 ± 0.57
NS, St. 2	58.1 ± 1.8	8.9 ± 0.8	6.53 ± 0.41
WS, St. 3	59.9 ± 1.8	9.8 ± 0.6	6.12 ± 0.31
BS, St. 4	75.2 ± 1.3	13.6 ± 1.0	5.57 ± 0.35
BS, St. 5	62.1 ± 0.5	14.2 ± 0.4	4.38 ± 0.13
BS, St. 6	90.3 ± 0.4	17.5 ± 0.4	5.16 ± 0.10

Baffin Sea (BfS), Norwegian Sea (NS), White Sea (WS), and Barents Sea (BS).

**Table 3:** The concentrations of tested elements (mg/kg DW) in samples of Arctic *F. distichus* (mean ± SD, n = 3).

Element	LOQ	Mean ± sd	Range (min–max)	St. 1	St. 2	St. 3	St. 4	St. 5	St. 6
Al	1.6	68.9 ± 37.5	33.3–126.2	58.7 ± 4.8	38.0 ± 2.8	103.4 ± 4.0	33.3 ± 1.2	53.7 ± 4.3	126.2 ± 18.9
As	6.3	32.4 ± 15.4	19.2–58.5	19.2 ± 3.3	27.2 ± 1.7	21.6 ± 0.7	58.5 ± 0.7	43.4 ± 2.9	24.7 ± 0.9
Ba	0.016	13.0 ±7.7	7.3–28.0	10.4 ± 0.2	7.3 ± 1.6	28.0 ± 0.2	14.0 ± 0.6	10.4 ± 0.2	7.9 ± 0.2
Ca	1.9	14,774 ± 5565	9490–25,476	15,029 ± 177	13,816 ± 509	12,037 ± 268	12,795 ± 255	25,476 ± 580	9490 ± 17
Cd	0.23	<LOQ	<LOQ	<LOQ	<LOQ	<LOQ	<LOQ	<LOQ	<LOQ
Co	0.12	1.8 ± 2.3	0.6–6.5	0.96 ± 0.02	0.71 ± 0.06	6.46 ± 0.02	1.03 ± 0.05	1.31 ± 0.01	0.62 ± 0.01
Cr	0.13	<LOQ	< LOQ	<LOQ	<LOQ	<LOQ	<LOQ	<LOQ	<LOQ
Cu	0.37	1.7 ± 1.1	0.6–3.2	3.23 ± 0.12	1.46 ± 0.09	2.86 ± 0.18	1.11 ± 0.24	0.97 ± 0.04	0.60 ± 0.01
Fe	0.098	214 ± 190	74–562	73.8 ± 10.7	112 ± 21	562 ± 15	97.1 ± 6.6	132 ± 19	310 ± 10
Mg	1.7	9304 ± 700	8400–10,222	10222 ± 124	9510 ± 31	9904 ± 171	9056 ± 20	8731 ± 67	8400 ± 28
Mn	0.058	45.8 ± 27.8	15.3–90.1	59.7 ± 1.7	15.3 ± 1.4	90.1 ± 4.4	32.7 ± 0.6	54.3 ± 0.3	22.8 ± 0.2
Ni	0.3	10.2 ± 1.1	<LOQ–10.9	<LOQ	<LOQ	<LOQ	9.5 ± 0.09	10.9 ± 0.04	<LOQ
Pb	4.6	<LOQ	<LOQ	<LOQ	<LOQ	<LOQ	<LOQ	<LOQ	<LOQ
Rb	0.55	36.4 ± 12.8	22.5–54.1	54.1 ± 1.5	35.8 ± 0.7	49.8 ± 1.2	28.9 ± 0.9	22.5 ± 0.6	27.3 ± 3.0
Sr	0.026	875 ± 130	704–1051	828 ± 35	833 ± 21	1009 ± 30	1051 ± 32	828 ± 8	704 ± 9
Zn	0.17	33.8 ± 8.1	26.9–44.6	27.3 ± 1.0	27.7 ± 1.7	33.1 ± 0.5	42.9 ± 0.9	44.6 ±0.3	26.9 ±1.0

LOQ—limit of quantification; St. 1–St. 6—the sampling stations.

**Table 4:** Element concentrations, their daily dose for *F. distichus* from different Arctic regions, and comparison with daily dose risk estimators for a 70-kg man and with nutritional requirements.

Element	Sampling Site with a Maximum Concentration	Mean–Max Concentration (mg/kg)	Single Dose for 3.3 g Consumption (mg/Day)	Daily Dose for 12.5 g Consumption (mg/Day)	Daily Dose from Risk Estimators	Daily Nutritional Requirements
Al	BS, St. 6	68.9–126.2	0.23–0.42	0.86–1.58	70 ^1^	10 ^5^
As (total)	BS, St. 4	32.4–58.5	0.11–0.19	0.41–0.73	0.15 ^1^ (inorganic)	5.0 ^6^
Ba	WS, St. 3	13.0–28.0	0.04–0.09	0.16–0.35	200	0.75 ^5^
Ca	BS, St. 5	14,774–25,476	49–84	185–318	2500 ^2^	1000 ^3^
Co	WS, St. 3	1.8–6.5	0.006–0.021	0.023–0.081	30 ^5^	10 ^5^
Cu	BfS, St. 1	1.7–3.2	0.006–0.011	0.021–0.040	5 ^2,5^	0.9 ^4^/1.0 ^5^
Fe	WS, St. 3	214–562	0.71–1.86	2.68–7.03	45 ^5^	10 ^3,5^
Mg	BfS, St. 1	9304–10,222	31–34	116–128	800 ^5^	400 ^5^
Mn	WS, St. 3	45.8–90.1	0.15–0.30	0.57–1.13	11 ^5^	2.7 ^3^/2.0 ^5^
Ni	BS, St. 5	3.6–11.0	0.012–0.036	0.045–0.14	20 ^5^	0.2 ^5^
Rb	BfS, St. 1	36.4–56.1	0.12–0.18	0.45–0.68	200	2.2 ^5^
Sr	BS, St. 4	875–1051	2.89–3.47	10.9–13.1	11 ^5^	1.9 ^5^
Zn	BS, St. 5	33.8–44.6	0.11–0.15	0.42–0.56	25 ^2^/40 ^5^	12 ^3,5^

^1^ PTWI: Provisional tolerable weekly intake; ^2^ UL: Tolerable upper intake level; ^3^ PRI: Population reference intake; ^4^ AI: Adequate intake; ^5^ [43]; ^6^ [80].

## Data Availability

All data generated or analyzed during this study are included in this published article.
